# The fifty billion dollar question: does formula cause necrotizing enterocolitis?

**DOI:** 10.1038/s41372-025-02277-2

**Published:** 2025-03-26

**Authors:** Mark A. Underwood

**Affiliations:** Emeritus Professor of Pediatrics, University of California Davis, Sacramento, CA USA

**Keywords:** Risk factors, Intestinal diseases, Outcomes research

## Abstract

The question of whether preterm infant formulas cause necrotizing enterocolitis (NEC) is the subject of multiple lawsuits and has daily relevance in the care of preterm infants. Research supporting the hypothesis that toxic components in infant formula cause NEC is limited to preclinical data while data from human infants are lacking. Human milk should be the first choice for most preterm infants, however, preterm infant formula is at times a critical alternative. It is the absence of human milk that increases NEC risk rather than toxic components in preterm infant formula.

## Introduction

Two separate lawsuits alleging that preterm infant formula caused necrotizing enterocolitis (NEC) in preterm infants resulted in awards of $60 M and $495 M against two formula companies in the United States in 2024. A third trial completed in October 2024 did not result in a judgment against a formula company. With about 1000 similar cases already filed, the potential impact of litigation on formula manufacturers and infant nutrition is significant [1]. NEC is a potentially devastating inflammatory intestinal disease with a global incidence of 7% among infants with birth weight less than 1500 g with high variability across studies [2]. Several studies have shown higher incidence among the smallest infants including those with lower gestational age, lower birth weight, and those who are small for gestational age [3–5]. Mortality rates are as high as 30%, and long-term consequences among survivors include short gut syndrome, malnutrition, chronic renal insufficiency, and poor neurodevelopmental outcomes. In multiple studies, the primary risk factor for NEC is preterm birth with the smallest and most preterm infants at the highest risk. The purpose of this review is to assess the evidence for a causal role of infant formula in the development of NEC.

### Pathogenesis

NEC is likely not a single discreet entity, but rather a final pathway to intestinal inflammation and necrosis with multiple starting points. To switch analogies, NEC is likely a perfect storm at the intersection of one or more of the following: immature intestinal anatomy, physiology, immune responses, intestinal dysbiosis, and inconsistent intestinal perfusion and oxygenation. In many cases, the poorly regulated inflammatory response begins *in utero*.

### Risk factors

In addition to preterm birth and low birth weight, several risk factors for NEC have been identified with some variation between studies. One approach is to consider prenatal, perinatal, and postnatal factors recognizing some degree of overlap. Table 1 presents odds ratios from recent papers for some NEC risk factors [6–13], and Table 2 presents odds ratios for some NEC protective factors in preterm infants [11].

**Table 1:** Risk factors for NEC.

Factor	OR or adjusted OR (95% CI)
Prenatal	
Small for gestational age [6]	OR 2.27 (1.35, 3.81), *p* = 0.002
Small for gestational age (sNEC) [7]	OR 1.68 (1.17, 2.36), *p* = 0.004
Gestational diabetes mellitus [6]	OR 1.57 (1.04, 2.38), *p* = 0.033
Gestational diabetes mellitus [8]	OR 3.08 (1.73, 5.48), *p* < 0.001
Cigarette smoking [9]	aOR 2.86 (1.14, 7.14), *p* = 0.02
Perinatal	
Pre-eclampsia [6]	OR 2.53 (1.27, 5.05), *p* = 0.008
Placental abruption^a^ [10]	aOR 1.2 (1.1, 1.3), *p* < 0.001
Chorioamnionitis [13]	aOR 1.12 (1.02, 1.15), *p* = 0.01
Premature rupture of membranes [9]	aOR 3.51 (1.77, 6.98), *p* < 0.001
Rupture of membranes > 7 days (sNEC) [12]	aOR 6.93 (1.56, 30.7), *p* = 0.041
Gestational age less than 29 weeks [6]	OR 28.1 (13.69, 57.51), *P* < 0.001
Neonatal asphyxia [8]	OR 2.46 (2.07, 2.93), *p* < 0.001
Meconium-stained amniotic fluid [8]	OR 3.14 (1.64, 6.01), *p* < 0.001
Postnatal	
TRIPS score 10–20 [6]	OR 1.61 (1.07, 2.41), *p* = 0.022
TRIPS score > 20 [6]	OR 2.57 (1.30, 5.05), *p* = 0.006
Hypotension (sNEC) [7]	OR 1.49 (1.18, 1.89), *p* = 0.001
Intraventricular hemorrhage (sNEC) [7]	OR 1.63 (1.30, 2.05), *p* < 0.001
Hemodynamically significant patent ductus arteriosus [6]	OR 3.44 (2.04, 5.80), *p* < 0.001
Patent ductus arteriosus [8]	OR 3.10 (1.93, 4.98), *p* < 0.001
Early or late onset sepsis [6]	OR 4.31 (2.52, 7.37), *p* < 0.001
Sepsis (in small for gestational age babies) [11]	OR 2.40 (1.27, 4.53, *p* = 0.007
Sepsis [8]	OR 3.91 (3.37, 4.55), *p* < 0.001
Pneumonia [8]	OR 6.17 (3.98, 9.57), *p* < 0.001
Red blood cell transfusion [6]	OR 3.25 (1.87, 5.65), *p* < 0.001
Red blood cell transfusion [8]	OR 2.41 (1.97, 2.95), *p* < 0.001
Anemia (in small for gestational age babies) [11]	OR 2.21 (1.17, 4.20), *p* 0.015
Severe anemia [8]	OR 2.86 (2.06, 3.99), *p* < 0.001
Hypoxemia [6]	OR 3.18 (2.05, 4.93), *p* < 0.001
Respiratory distress syndrome [6]	OR 2.80 (1.70, 4.61), *p* < 0.001
Respiratory distress syndrome [8]	OR 3.28 (2.23, 4.85), *p* < 0.001
Respiratory failure [8]	OR 7.51 (1.60, 35.10), *p* = 0.01
Congenital heart disease [8]	OR 4.80 (3.00, 7.68), *p* < 0.001
Acid blocking agents [6]	OR 3.49 (1.22, 10.00), *p* = 0.02
Antibiotics [8]	OR 2.12 (1.18,3.81), *p* = 0.01
Severe feeding intolerance [6]	OR 6.75 (4.17, 10.91), *p* < 0.001
Hypoproteinemia [8]	OR 2.80 (1.78, 4.41), *p* < 0.001
Umbilical arterial catheter > 5 days (sNEC) [12]	aOR 3.8 (1.05, 13.7), *p* = 0.041

*sNEC* surgical NEC, *TRIPS* transport risk index of physiologic stability.

^a^Only in infants with birth weight > 1500 g.

**Table 2:** Protective factors for NEC.

Factor	OR or adjusted OR (95% CI)
Prenatal	
Normal body mass index [9]	aOR 0.11(0.02, 0.58), *p* = 0.009
Perinatal	
Antenatal corticosteroids [8]	OR 0.38 (0.24, 0.60), *p* < 0.001
Antenatal corticosteroids (sNEC) [7]	OR 0.80 (0.64, 0.99), *p* = 0.044
Postnatal	
Breast milk feeding [6]	OR 0.26 (0.12, 0.57), *p* < 0.01
Breast milk feeding [8]	OR 0.31 (0.16, 0.62), *p* < 0.001
Probiotics [8]	OR 0.36 (0.25, 0.53), *p* < 0.001
Probiotics (in small for gestational age babies) [11]	OR 0.49 (0.30, 0.80), *p* = 0.004

*sNEC* surgical necrotizing enterocolitis.

### Unpasteurized human milk

The studies in Table 2 are among several that have shown a lower incidence of NEC in preterm infants receiving unpasteurized mother’s own milk. Human milk is a complex tissue containing an array of bioactive components that shape the infant's intestinal microbiota and impact the developing infant's immune system. Table 3 summarizes many of these components and includes potential protective mechanisms [14–26]. Studies comparing raw mother’s milk (never frozen or pasteurized) to mother’s own milk that has been frozen or pasteurized in preterm infants have been small with limited evidence for benefit [27].

**Table 3:** Some of the bioactive components of human milk [references 14–26].

	Growth factor	Enhanced barrier function	Prebiotic	Anti-inflammatory	Antimicrobial	Anti-oxidant	Degraded by pasteurization	Degraded by freezing
Immunoglobulins			X		X		X	
Lactoferrin	X	X		X	X	X	X	X
Lysozyme					X		X	
Alpha lactalbumin					X		X	
Human milk oligosaccharides		X	X	X	X	X		
Bile salt stimulating lipase					X		X	
Human milk fat globule membrane proteins		X			X	X	X	
Long chain PUFAs				X			X	
Epidermal growth factor	X			X			X	
Insulin-like growth factors 1/2	X						X	
Erythropoietin	X						X	
Insulin	X						X	
Adiponectin				X			X	
Melatonin		X		X		X	X	
Vascular endothelial growth factor	X						X	
Glycosaminoglycans		X	X	X	X			
Glutathione peroxidase						X	X	X
Superoxide dismutase						X	X	X
microRNAs		X		X			X	
White blood cells				X	X		X	
Stem cells	X						X	
Bacteria				X			X	
Exosomes	X			X	X		X	

*PUFAs* polyunsaturated fatty acids.

### Pasteurized donor human milk

The 2024 Cochrane review of pasteurized donor human milk (generally a pooled product from multiple donors) vs infant formula for preterm infants included 12 trials from Europe and North America (five of these studies were conducted more than 40 years ago). The meta-analysis found that donor human milk reduces the risk of NEC (RR 0.53, 95% CI 0.37, 0.76) but does not alter the risk of late-onset sepsis or death [28]. Donor milk is generally lower in protein content than mother’s own milk. Pasteurization is effective at destroying potentially pathogenic bacteria and viruses but also denatures bioactive proteins and peptides. Table 3 also includes the impact of pasteurization on bioactive human milk components.

### Fortifying human milk

Human milk is often fortified for premature infants to improve growth and minimize fluid overload. Commercially available fortifiers are either made from bovine milk or donor human milk. In recent years, a variety of commercially available bovine fortifiers have become available including acidified vs non-acidified, intact vs hydrolyzed protein, and powder vs liquid. Unfortunately few studies have been performed comparing feeding tolerance and growth among these products [29]. A meta-analysis of clinical trials comparing bovine to human milk-based fortifiers included 681 infants with gestational age less than 28 weeks or birth weight less than 1500 g and found decreased mortality with the human milk-based fortifier, but no differences in NEC, sepsis, retinopathy of prematurity or bronchopulmonary dysplasia [30]. A retrospective review of 98 preterm infants found a higher percentage of episodes of hypoglycemia in infants receiving a human milk-based fortifier compared to a bovine milk-based fortifier [31].

### Preterm infant formula

Formulas for preterm infants were developed in the 1970s and 1980s to address two concerns: poor growth in preterms receiving unfortified mother’s milk and the HIV epidemic (which resulted in the closure of most human milk banks) [32]. The first studies comparing risk of NEC in infants based on diet were published in the early 1990s with most but not all showing lower rates with human milk than formula [28, 33–35]. The central question is whether this difference is due to protective factors in human milk that are not in the formula (Table 3) or whether preterm infant formulas contain one or more toxic components that trigger NEC. Preclinical studies provide insights and hypotheses that help to address this question, including evidence for potential injury from the key macronutrients protein, carbohydrate, and fat.

#### Bovine milk protein

The protein source for preterm infant formulas is bovine milk. Allergy to and intolerance of bovine milk protein occurs in 2% of term infants with symptoms ranging from mild to severe with enterocolitis and shock [36]. In preterm infants, bovine protein intolerance can present with hematochezia and even pneumatosis intestinalis making the distinction between NEC and bovine protein intolerance difficult at times [37]; generally, preterm infants with the latter are less ill, more likely to have recurrent episodes, have higher eosinophil counts and lower procalcitonin levels and demonstrate recurrence of hematochezia with re-introduction of intact bovine milk protein [38]. Many animal models include bovine formula as a trigger which is necessary but not sufficient to induce a NEC-like illness. In preterm piglets, methods of processing bovine protein and duration of storage influence the impact of formula feeding on intestinal development and NEC-like illness [39]. In mice, NEC severity is similar whether the mice receive an amino acid formula (no intact proteins or peptides) or a preterm formula with intact bovine protein [40]. Data from preterm infants suggesting that bovine milk protein is a trigger for NEC are lacking. It has been proposed that incomplete digestion of dietary protein in the preterm intestine stimulates dysbiosis, inflammation, and NEC, however proteomic analysis comparing stools from preterm infants just prior to NEC onset to controls did not show differences suggestive of maldigestion [41]. If intact bovine proteins or peptides commonly trigger NEC, one would expect an extensively hydrolyzed formula to decrease NEC risk. A recent meta-analysis of 11 small clinical trials (665 preterm infants) comparing extensively hydrolyzed formula to standard formula found no difference in NEC [42]. Human milk contains intact bovine protein unless this is removed from the maternal diet. In a small study, 120 mothers providing milk to their preterm infants were randomized to dairy-free diet vs standard diet with no difference in stage 2–3 NEC between groups [43].

#### Carbohydrate

The carbohydrates in human milk consist of lactose and human milk oligosaccharides (HMOs). Lactose is a disaccharide that requires enzymatic digestion by lactase to release the monosaccharides glucose and galactose. Lactase activity in preterm infants is lower than for term infants, but lactose intolerance in preterm infants is uncommon. HMOs are abundant in human milk with a wide variety of structures and sizes and yet are not digestible by the infant due to lack of the glycosidases necessary to digest them. The question as to why a mother expends significant energy to synthesize these molecules which have no nutritional value for her infant is a compelling one. The discovery that only a limited number of intestinal microbes (*Bifidobacterium* and *Bacteroides* species) are able to digest and transport HMOs into their cytoplasm suggests that one of the primary roles of HMOs is to shape the developing intestinal microbiome of the infant [44]. In animal models, HMOs also stimulate mucin production [45] and have anti-inflammatory properties [46].

The primary carbohydrates in preterm infant formulas are maltodextrin and corn syrup solids due to their rapid digestibility and high glycemic index. Both are polymers of glucose with predominantly alpha 1,4 linkages that are digested by maltase which is expressed at higher levels in the preterm infant than lactase. In the preterm piglet, maltase expression is low and administration of maltodextrin triggers maldigestion and a NEC-like illness [47]. It has been proposed that incomplete digestion of dietary maltodextrin in the preterm intestine stimulates dysbiosis, inflammation, and NEC, however, a randomized trial in 306 preterm infants comparing maltose-containing formula to lactose-containing formula showed no differences in NEC incidence but less feeding intolerance in the maltose-containing formula group [48]. If maldigestion of maltodextrin were common in formula-fed preterm infants, one would expect to see frequent feeding intolerance, diarrhea, and poor growth which is not the case.

#### Fat

In both rodent and piglet models, manipulating the fat content of the diet (percentage of total energy, percentage of saturated fatty acids, and percentage polyunsaturated fatty acids (PUFAs)) alters the intestinal microbiota [49]. In a study designed to explore mechanisms by which dietary fat content might influence the risk of inflammatory bowel disease, 4-week-old weaned mice demonstrated increased Enterobacteriaceae and increased intestinal inflammation with a diet rich in n-6 PUFAs and increased bifidobacteria, lactobacilli and enterococci but increased sepsis with a diet rich in n-3 PUFAs [50]. In an established rat NEC model, a diet rich in PUFAs resulted in less NEC than a standard formula without added PUFA [51].

Of the macronutrients in human milk, fat is the most variable, both between women and in a given woman over time. This variation includes fat content (5-fold differences) and percentages of individual fatty acids (e.g., 20-fold differences in DHA) [49]. In addition, freezing/thawing and exposure to plastic surfaces (e.g., bottles and tubing) impact fat content. This variability in the amount of fat delivered makes it difficult to determine the impact of dietary fat on NEC risk in preterm infants. It has been proposed that maldigestion and malabsorption of fat in the formula-fed preterm gut is a trigger of NEC, however, a small study found little difference in fat absorption between term and preterm infants and between formula and human milk-fed infants [52]. If fat maldigestion or malabsorption were common in formula-fed preterm infants, one would expect to commonly see diarrhea and poor weight gain which is not the case.

#### Osmolality

It is striking that in spite of all the cellular and bioactive components in human milk, its osmolality is fairly low. Fortification of human milk and feeding of more concentrated infant formulas are common in preterm infants, particularly those with poor growth. In addition to increasing calories, fortification increases osmolality. Extensive studies of changes in osmolality with a wide variety of fortification strategies show increases from a baseline of 284–300 mOsm/kg in unfortified human milk to greater than 700 mOsm/kg in some of the fortified products [53–55]. Osmolality of commercial products is influenced in part by the degree of hydrolysis of the protein component with elemental formulas having the highest osmolality [54]. It has been hypothesized that higher osmolality feeding injures the gut leading to increased permeability and inflammation potentially triggering NEC. A recent meta-analysis of human and animal studies of osmolality included 10 human studies (7 found no differences in adverse events based on feeding osmolality, 1 found delayed gastric emptying with hyperosmolar feeds, 1 found increased NEC with hyperosmolar feeds and 1 found increased NEC with the lower osmolar feeds) and 6 animal studies (none found increased NEC incidence with higher osmolality feeds) [56]. In summary, it is clear that fortification of human milk increases its osmolality, but evidence that higher osmolality increases NEC risk is lacking with some experts calling for more research to reassess the safe upper threshold for osmolality in preterm infants [54].

### Diet and Intestinal dysbiosis

In term infants, the primary factors influencing the composition of the intestinal microbiota are diet, mode of delivery and antibiotic exposure. The intestinal microbiota of preterm infants differs substantially from that of term infants and is most heavily impacted by post-menstrual age. A recent systematic review of research on the developing intestinal microbiota in preterm infants found a variety of perinatal, physiological, pharmacological, dietary, and environmental factors shape this community [57]. Since the publication of that meta-analysis, a randomized controlled trial found no significant differences in the fecal microbiota of preterm infants receiving mother’s milk supplemented with pasteurized donor human milk and human milk-based fortifier compared to mother’s milk supplemented with bovine formula and bovine milk fortifier [58]. These studies support the hypotheses that diet is only one of several factors impacting the intestinal microbiota in preterm infants, likely not among the primary factors, and that the protective benefit of unpasteurized human milk against NEC is not primarily related to its impact on the microbiota.

### Diet and intestinal perfusion

Intestinal perfusion is influenced by the vasodilatory molecule endothelial nitric oxide synthase (eNOS). In cultured endothelial cells, a common HMO, 2′fucosyllactose, induces eNOS expression and in wild-type mice, this same HMO is protective against NEC while this protective effect is not seen in mice deficient in eNOS or in wild-type mice given an eNOS inhibitor [59]. Splanchnic perfusion can be estimated with near-infrared spectroscopy. A small study found sustained decreases in splanchnic perfusion with preterm formula feeds, transient decreases with fortified human milk, and no decrease with unfortified mother’s milk [60], however, this study has been questioned given that the measurements were made at a postmenstrual age that is greater than when NEC typically occurs and that the groups were not well matched for gestational age, degree of anemia or presence of chronic lung disease [61]. The importance of intestinal perfusion in NEC pathogenesis is supported by pre-clinical models (e.g., of remote ischemic conditioning [62]), however, more studies are needed to understand perfusion’s role in NEC and how diet affects perfusion in the preterm infant.

### Prognostic studies

In causal inference, a confounder is a variable that impacts both the exposure and the outcome, creating a spurious association. Mediators, on the other hand, are both effects of the exposure and a cause of the outcome. Covariates are independent variables that explain some of the variability in the outcome variable. Distinguishing between confounders, mediators and covariates is not always straightforward, particularly when the data available come from case-control and cohort studies (as do all the data in Tables 1 and 2). For example, pre-eclampsia, maternal obesity, and maternal smoking have each been shown in some (but not all) studies to be associated with increased incidence of NEC [63–65], with decreased success in providing human milk [66–68], and with alterations in the composition of human milk [69–71] and are therefore important confounders or covariates. Multiple regression modeling that includes potential covariates is superior to simple regression (in which a single predictor variable is used to predict an outcome variable) in complex biological processes like NEC pathogenesis. Prognostic studies are the preferred approach to determining independent risk factors for complex disease and ideally are prospective and utilize multivariable analysis including multiple covariates. A recent review found few prognostic studies of NEC and did not identify infant diet as a significant prognostic factor [72].

### Expert opinion

A working group was organized by the National Advisory Council of Child Health and Human Development with the charge to assess the scientific evidence regarding enteral feeding practices and risk of NEC [73]. This working group identified many knowledge gaps and included 17 recommendations to address these gaps in their summary. The summary statements that are most relevant to the current question include the following from Box 2.1: “Available evidence supports the hypothesis that it is the absence of human milk—rather than the exposure to formula—that is associated with an increase in the risk of NEC,” and the following from page 22: “It is unknown whether human milk fortified with an HMF [human milk-based fortifier], compared to human milk fortified with a BMF [bovine milk-based fortifier], could decrease the risk of NEC. There are few rigorous studies that have addressed these questions, and the study numbers were too small to make definitive conclusions”. Following the release of this summary from the working group in September 2024, a joint statement was released from the Food and Drug Administration, the National Institutes of Health, and the Centers for Disease Control and Prevention including the following (emphasis in the original): “There are two key points about feeding practices and NEC: *(1) There is no conclusive evidence that preterm infant formula causes NEC; and (2) there is strong evidence that human milk is protective against NEC”* [74]. From the same joint statement (emphasis in the original): “While mother’s milk is the preferred source of nutrition—with pasteurized donor human milk as a next best alternative—preterm infant formulas are a critically important option for premature infants. These formulas can be critical for premature infants for whom parental or donor milk is not an option, or where a supplement to parental or donor milk is necessary for the health of the infant. *For infants where the supply of human milk is insufficient, these formulas are part of the standard of care for premature infants*” [74].

## Conclusion

Human milk is a complex fluid produced at great expense by the mother providing both nutrition and bioactivity to her infant. The scientific evidence supports a role of human milk in decreasing the risk of NEC, but not the role of infant formula in causing NEC. Openness and transparency in communications with parents of preterm infants about what we know and don’t know about the benefits of human milk [75] and overcoming obstacles to the provision of mother’s milk to preterm infants [76] (implementation of proven strategies [76], additional research into increasing abundance and utilization of mother’s milk (e.g., well-designed trials of secretagogues), and novel funding methods for human milk (e.g., the Access to Donor Human Milk Act of 2023 and/or direct incentives to mothers of preterm infants)) are essential to progress towards “a world without NEC” [77].
